# GluN2A: A Promising Target for Developing Novel Antidepressants

**DOI:** 10.1093/ijnp/pyae037

**Published:** 2024-08-26

**Authors:** Gang Wang, Wang Qi, Qiu-Hua Liu, Wei Guan

**Affiliations:** Department of Hepatobiliary Surgery, Zhangjiagang Hospital affiliated to Soochow University/The First People’s Hospital of Zhangjiagang City, Zhangjiagang, China; Department of Pharmacology, The First People’s Hospital of Yancheng, Yancheng, China; Department of Hepatobiliary Surgery, Zhangjiagang Hospital affiliated to Soochow University/The First People’s Hospital of Zhangjiagang City, Zhangjiagang, China; Department of Pharmacology, Pharmacy College, Nantong University, Nantong, China

**Keywords:** Depression, GluN2A, depressive-like behavior, neurogenesis, antidepressants

## Abstract

**Background:**

Depression is a heterogeneous disorder with high morbidity and disability rates that poses serious problems regarding mental health care. It is now well established that N-methyl D-aspartate receptor (NMDAR) modulators are being increasingly explored as potential therapeutic options for treating depression, although relatively little is known about their mechanisms of action. NMDARs are glutamate-gated ion channels that are ubiquitously expressed in the central nervous system (CNS), and they have been shown to play key roles in excitatory synaptic transmission. GluN2A, the predominant Glu2N subunit of functional NMDARs in neurons, is involved in various physiological processes in the CNS and is associated with diseases such as anxiety, depression, and schizophrenia. However, the role of GluN2A in the pathophysiology of depression has not yet been elucidated.

**Methods:**

We reviewed several past studies to better understand the function of GluN2A in depression. Additionally, we also summarized the pathogenesis of depression based on the regulation of GluN2A expression, particularly its interaction with neuroinflammation and neurogenesis, which has received considerable critical attention and is highly implicated in the onset of depression.

**Results:**

These evidence suggests that GluN2A overexpression impairs structural and functional synaptic plasticity, which contributes to the development of depression. Consequently, this knowledge is vital for the development of selective antagonists targeting GluN2A subunits using pharmacological and molecular methods.

**Conclusions:**

Specific inhibition of the GluN2A NMDAR subunit is resistant to chronic stress-induced depressive-like behaviors, making them promising targets for the development of novel antidepressants.

## INTRODUCTION

Depression is one of the most prevalent neuropsychiatric disorders, characterized by low mood, anhedonia, and even suicidal ideation, which negatively influences people’s physical and mental health, functioning, and also their quality of life (Wang et al., 2021a). According to World Health Organization reports, approximately 350 million people worldwide suffer from depression; this number is increasing and is the leading cause of disability (Koopman and El Aidy, 2017). Although increasing evidence suggests that a combination of genetic predisposition and life events, mainly exposure to stress, is a common etiological factor in the development of depression (Torres-Berrío et al., 2019), the etiology underlying this disorder still remains unclear. In recent decades, considerable progress has been made in the research and development of treatments for depression in several settings, including antidepressant medications and psychotherapy (Cuijpers et al., 2019). The majority of patients with depressive symptoms improve during treatment, but approximately 20%–40% of patients do not exhibit a clinical response to current treatment with antidepressants (Touloumis, 2021). In addition, limited research has investigated whether clinicians around the world have found diagnostic criteria for depression that are useful with diverse populations, while a significant number of patients with depression are undiagnosed or misdiagnosed. Thus, exploring the more precise pathogenesis of depression is necessary to develop safer and more effective therapeutic strategies.

The pathophysiology of depression is complex and involves multiple biological factors, including monoamine neurotransmitter disturbances, neuroinflammation, dysregulation of the hypothalamus-pituitary-adrenal axis, alterations in intestinal flora composition, and neuroplasticity (Yu et al., 2023). Among these, the monoamine hypothesis, in conjunction with the efficacy of antidepressants targeting monoamine systems, has long been the central topic of depression research. However, it falls short in explaining the disease’s onset and progression, leaving treatments often less than optimal, and many patients with major depressive disorder (MDD) remain refractory to monoamine-based antidepressants (Kim et al., 2023). Given that, the glutamate hypothesis is gaining attention as a novel hypothesis that can overcome monoamine restrictions (Kim et al., 2023). A growing body of preclinical research suggests that the brain glutamate system is a target of traditional monoaminergic-based antidepressants and may be involved in the pathophysiology of depression (Catena-Dell’Osso et al., 2013). Regulation of glutamate levels is pivotal for normal brain function (Palop et al., 2006), whereas elevated concentrations of glutamate cause excitatory signaling and cytotoxicity, leading to neuronal damage and death (Soni et al., 2014; O’Donovan et al., 2017). Recent studies have shown that rapid-acting antidepressants regulate glutamate receptors, reducing the function of NMDARs, glutamate release, and synaptic transmission (Musazzi et al., 2013). NMDARs are ligand-gated ion channels required for excitatory neurotransmission and plasticity of excitatory synapses (Yashiro and Philpot, 2008). In addition, they have many binding sites for endogenous molecules and drugs and are permeable to Na^+^, K^+^, and Ca^2+^ (Yang et al., 2005). A growing body of preclinical research suggests that NMDARs are able to conduct high amounts of Ca^2+^ ions, contributing to elevated intracellular Ca^2+^ concentrations that are important for synaptic plasticity (Myers et al., 2016), while the enhanced activity of NMDAR leads to Ca^2+^ overload and Ca^2+^ influx into postsynaptic neurons that generate a slow excitotoxicity process (Thakral et al., 2023). Therefore, dysfunction of NMDAR in the CNS has been proved to be pivotal in the pathophysiology of depression and the mechanism of action of antidepressant treatments (Berman et al., 2000).

It is noteworthy that some recent evidence has suggested that GluN2A-containing NMDARs are thought to play crucial roles in neuronal plasticity and pathological conditions, such as depression (Francija et al., 2019). For example, Francija et al. showed that the disruption of the NMDAR GluN2A subunit in wild-type (WT) mice abolished inflammation-induced depression (Francija et al., 2019), indicating that the GluN2A subunit is involved in lipopolysaccharide (LPS)-induced depressive-like behavior. Moreover, Zanos et al. demonstrated that ketamine requires the pharmacological activation of the NMDAR subunit GluN2A to exert its antidepressant behavioral actions, whereas ketamine-induced antidepressant-relevant behavioral actions are blocked by GluN2A-preferring NMDAR antagonists (Zanos et al., 2023). These findings indicate that GluN2A activity mediates the antidepressant actions of ketamine. However, Wong et al. reported inconsistent and contradictory results and found no connection between the GluN2A subunit and long-term depression (LTD) (Wong and Gray, 2018). Regarding these inconsistent results, the exact mechanism underlying the role of GluN2A in depression is largely unknown despite a vast amount of past research. Therefore, in this review, we provide an overview of the pathophysiology of depression and focus on describing the mechanisms of action of GluN2A in depression based on these neurobiological underpinnings. The elucidation of GluN2A function is expected to further our understanding of the pathophysiology of depression and lead to the discovery of new therapeutic targets for depression.

## NMDARs

### Overview of NMDARs

**Figure 1.**
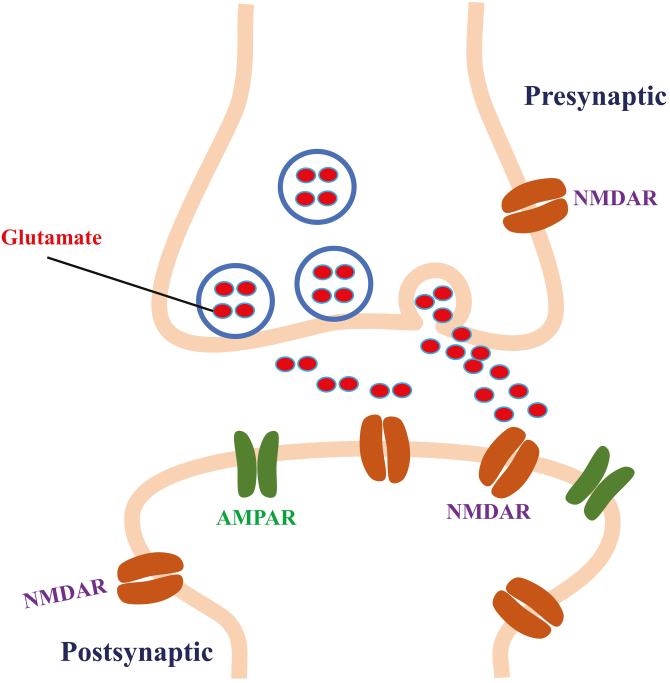
The function of N-methyl D-aspartate receptors (NMDARs) in synaptic plasticity. NMDARs are found both pre- and postsynaptically. It should be noted that at the resting membrane potential, the NMDAR channel is blocked by Mg^2+^, but this blockage can be released by the depolarization that accompanies rapid activation of the α-amino-3-hydroxy-5-methyl-4-isoxazolepropionic acid receptor (AMPAR). Then the presynaptic glutamate is released and combines with NMDARs, and the influx of Ca^2+^ ion is allowed to pass through the NMDARs.

NMDARs are ligand- and voltage-gated ionotropic glutamate receptors belonging to a subclass of glutamate receptors that promote Ca^2+^ and Na^+^ influx and play important roles in synaptic function and plasticity (Chen et al., 2020; Egbenya et al., 2021). It should be noted that at the resting membrane potential, the NMDAR channel is blocked by Mg2+, but this blockage can be released by the depolarization that accompanies rapid activation of the α-amino-3-hydroxy-5-methyl-4-isoxazolepropionic acid receptor (AMPAR) (Huganir and Nicoll, 2013). Subsequently, presynaptic glutamate is released and combines with NMDARs, and the influx of Ca^2+^ ions is allowed to pass through and propagate the action potential through NMDARs (Figure 1) (Mayer et al., 1984; Cotman et al., 1988). In addition to the location of NMDARs in the postsynaptic membrane (Duguid and Sjöström, 2006), the presence of NMDARs has long been described in the presynaptic membrane of several brain areas, such as the cortex (Berretta and Jones, 1996) and hippocampus (Siegel et al., 1994). In the postsynaptic membrane, NMDARs are found in synaptic, extrasynaptic, and perisynaptic positions and perform different functions (Shipton and Paulsen, 2014).

### NMDARs’ Subunit Composition and Subtype Selectivity

**Figure 2.**
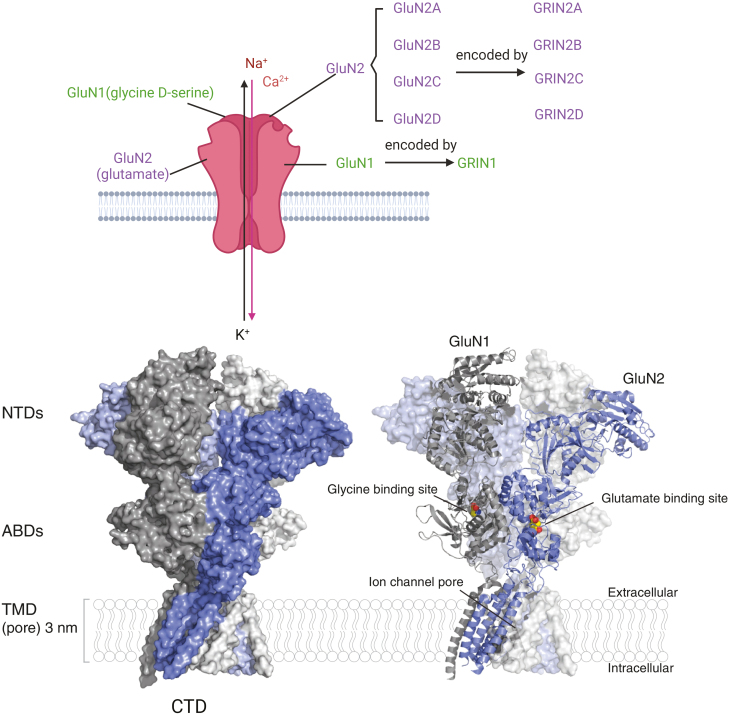
Structure of an NMDAR. Upper panel: Most of NMDARs are heterotetramers containing various subunits, including 2 obligatory GluN1 subunits and 2 regulatory subunits GluN2 (GluN2A-D). The GluN1 subunit is encoded by the GRIN1 gene, and the GluN2 subunits (GluN2A-D) are encoded sequentially by the GRIN2A-D genes. Down panel: X-ray and crystal structure of the hetero-tetrameric GluN1/GluN2 receptor. The 2 GluN1 subunits are represented in different shades of grey and the GluN2 subunits in different shades of blue. NMDARs comprise 4 domains: a large N-terminal domains (NTDs), a bilobed agonist-binding domains (ABDs), a pore-forming transmembrane domain (TMD), and an intracellular carboxyl-terminal domain (CTD) (McBain and Mayer, 1994). In addition, the figure also shows the location of the orthosteric site in GluN1 (bound to glycine) and GluN2 (bound to glutamate) and of the ion channel pore site in the transmembrane region. The down panel was adapted from Zhu et al. (Zhu and Paoletti, 2015). The figure was generated using BioRender (Agreement number: ZO272TFPPG).

**Figure 3.**
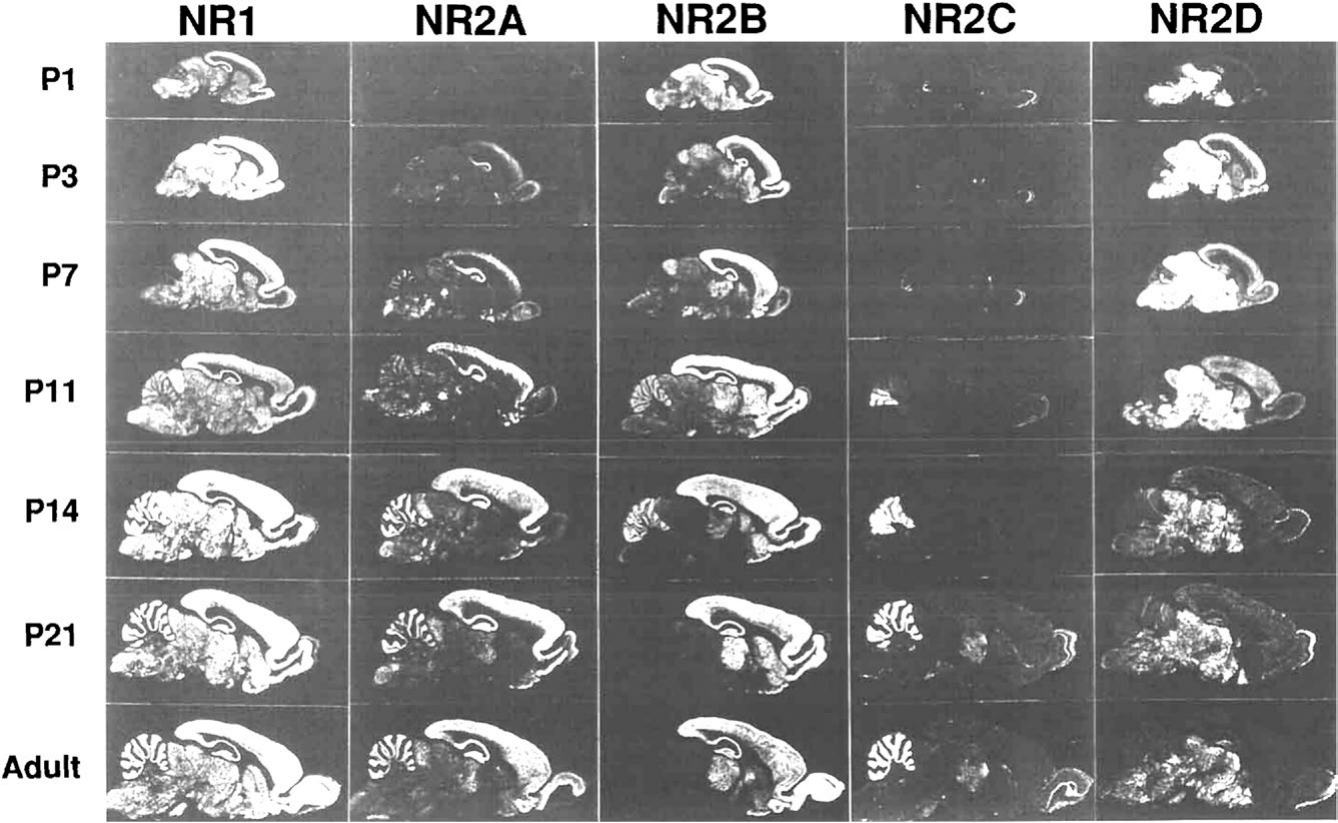
Autoradiograms obtained by in situ hybridizations of oligonucleotide probes to parasagittal sections of rat brains at the indicated postnatal (P) days reveal distinct regional and developmental expression of GluN2 subunits. Specifically, GluN2A progressively increased after birth in the brain until adulthood (predominantly in the forebrain), while GluN2B was widely distributed at an early stage and its expression peaks around postnatal day 7 (Akazawa et al., 1994). At this stage, there was a sharp rise in GluN2A expression, while the expression of GluN2B gradually remained at high levels and become confined to areas of the forebrain28, which was known as the “developmental GluN2B-GluN2A switch.” The figure was adapted from Akazawa et al. (Akazawa et al., 1994).

NMDARs are heterotetramers containing various subunits, including 2 obligatory GluN1 subunits with 8 splice variants, regulatory subunits GluN2 (GluN2A-D), and GluN3 (GluN3A-B) (Gray et al., 2011). Most of the actions reported for NMDARs are mediated by receptors comprising GluN1 and GluN2 subunits (Figure 2) (Salussolia et al., 2011). The GluN1 subunit is encoded by the GRIN1 gene. GluN2 subunits (GluN2A-D) are encoded sequentially by GRIN2A-D genes, and GluN3 subunits (GluN3A-B) are encoded by GRIN3A-B genes (Gray et al., 2015; Baucum, 2017). Previous studies have shown that the subunits assemble differently and perform various functions with distinct physiological functions (Al-Hallaq et al., 2007). For example, the GluN1 subunit, a mandatory subunit and a common feature of all NMDARs, is ubiquitously expressed from embryonic stage E14 to adulthood in rats (Akazawa et al., 1994). The GluN1 subunit has 8 different isoforms (a–h, with different splice variants of a single gene) that arise from the alternative splicing of 3 exons (Durand et al., 1992). The GluN2 subunit (GluN2A-D) has expression patterns that vary prominently in both time and location, that is, during development and in different regions (Akazawa et al., 1994). GluN2B/GluN2D and GluN3A/GluN3B are more abundant in early developmental stages, whereas GluN2A/GluN2C is more highly expressed in mature developmental stages (Akazawa et al., 1994; Monyer et al., 1994). GluN2A and GluN2B are the predominant GluN2 NMDAR subunits in the cortical and hippocampal regions of the brain (Akazawa et al., 1994; Monyer et al., 1994), although GluN2C and GluN2D are also present, particularly early in development, but in low quantities in adulthood (Monyer et al., 1994). Specifically, GluN2A progressively increases in the brain after birth until adulthood (predominantly in the forebrain), whereas GluN2B is widely distributed at an early stage and its expression peaks around postnatal day 7 (Figure 3) (Akazawa et al., 1994). At this stage, there is a sharp increase in GluN2A expression, while GluN2B gradually remains at high levels and becomes confined to areas of the forebrain (Akazawa et al., 1994), known as the “developmental GluN2B-GluN2A switch.” This indicates that synaptic NMDARs switch from a GluN2B-dominant type to a GluN2A-dominant type in response to neuronal activity and sensory experiences during development (Lau and Zukin, 2007). Notably, GluN2A- and GluN2B-containing NMDARs are predominantly found synaptically and extrasynaptically, respectively, with opposing effects on neurons (Vizi et al., 2013).

## Functional and Molecular Properties of GluN2A

### Expression Profile of GluN2A Subunit

GluN2A subunits, one of the most abundant and widely expressed NMDAR subunits in the adult brain, are instrumental in cerebral development and function and have always been a research hotspot. GluN2A subunit-containing NMDARs are highly expressed in the cerebral cortex and hippocampus and moderately expressed in the cerebellum, midbrain, brainstem, and striatum (Zhang and Luo, 2013). GluN2A is characterized by mature structures, and changes in GluN2A expression are associated with complex phenotypes that lead to complex neurological diseases. In addition to the brain tissue, the GluN2A subunit is found in peripheral tissues (Makhro et al., 2016). For example, Makhro et al. demonstrated that the expression of GluN2A was restricted to the atria of rats (Makhro et al., 2016) and that the activation of NMDARs was associated with the induction of tachycardia, sinus arrhythmia, and ischemia occurring within the physiological plasma concentration range for glutamate and glycine. Moreover, the GluN2A subunit has been detected in mouse bone marrow cells (Monyer et al., 1994) and glomerular cells in the kidney (Merle et al., 2003), and its overexpression has been detected in pancreatic cancer cells (Malsy et al., 2015).

### Expression, Functional, and Pharmacological Characteristics of GluN2A

**Figure 4.**
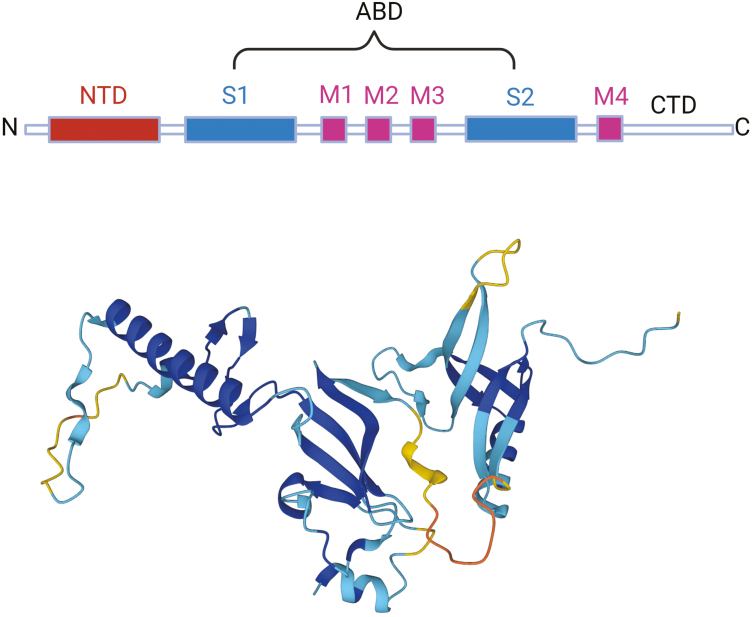
The architecture of GluN2A. Upper panel: GluN2A is made up of 4 domains: a large NTD, a bilobed ABD, a pore-forming TMD, and an intracellular CTD. The ABD is formed by the S1 and S2 segments of the polypeptide chain, which are separated by the M1, M2, and M3 segments. The TMD is formed by 3 transmembrane helices (M1, M3, and M4) and a reentrant loop (M2). Lower panel: the crystal structure of the GluN2A receptor (UniProt: A0A5A4LGJ9), it was created with the Alpha Fold Monomer v2.0 pipeline. Abbreviations: ABD, agonist-binding domain; CTD, carboxyl-terminal domain; NTD, N-terminal domain; TMD, transmembrane domain.

Related past studies suggested that GluN2A-containing NMDARs displayed faster kinetics and were implicated in the protective pathways by mediating long-term synaptic plasticity, while GluN2B-containing receptors displayed slower kinetics and increased neuronal vulnerability (Akashi et al., 2009; McQuail et al., 2016). Therefore, excessive NMDAR activation is harmful to neurons, and so is too little. GluN2A has an architecture similar to that of all glutamate receptor subunits and is composed of 4 domains: a large N-terminal domains (NTDs), a bilobed agonist-binding domain (ABDs), a pore-forming transmembrane domain, and an intracellular carboxyl-terminal domain (CTD) (Figure 4). Recently, several studies have demonstrated that GluN2A exists more abundantly at synaptic sites, desensitizes more, and takes less time to recover than GluN2B-containing receptors (Loftis and Janowsky, 2003), indicating that GluN2A is more flexible in regulating synaptic activity with these electrophysiological properties. Therefore, regarding the faster opening and closing velocities of GluN2A subunit-containing NMDARs, the high sensitivity to (S)-glutamate and glycine, the fast activation and deactivation kinetics compared with the corresponding NMDARs with GluN2B subunits (Goebel and Poosch, 1999), and the important characteristics of a switch from GluN2B- to GluN2A-containing NMDARs in synapses during the developmental period, GluN2A is unique among the NMDAR subtypes and has received increasing attention in many common human diseases. In addition to these pathophysiological processes, GluN2A-containing NMDARs are also involved in disorder like depression (Han et al., 2015), epilepsy (Zhu et al., 2004), and schizophrenia (Miyamoto et al., 2001). A clinical trial showed a de novo mutation of the GluN2A subunit (P1199Rfs*32) was identified in a male patient with epileptic encephalopathy and neurobehavioral changes (Vieira et al., 2024). Pharmacological experiments have shown that neuropeptide Y can inhibit seizures by downregulating the functional expression of GluN2A and GluN2B (Dong et al., 2013). In addition, mice lacking GluN2A have been found to exhibit several behavioral abnormalities related to schizophrenia, including hyperlocomotion and cognitive impairments (Miyamoto et al., 2001). These aforementioned data suggest that activation or inhibition of GluN2A-containing NMDAR is essential for the pathogenesis of epilepsy and schizophrenia. However, the role of GluN2A-containing NMDAR in depression is not well characterized. Therefore, to better understand the role of GluN2A-containing receptors in depression, we have focused on synaptic GluN2A-containing NMDARs, their role in synaptic plasticity, and their contribution to pathological plasticity as observed in depression. Moreover, we have discussed how GluN2A expression mediates the NMDAR activation-dependent effects of ketamine.

### Electrophysiological Properties of GluN2A Subunit

GluN2A receptors can be classified into 2 types: diheteromers and triheteromers. The most typical GluN2A receptors, diheteromeric GluN2A receptors consist of 2 GluN1 subunits and 2 GluN2A subunits (GluN1/GluN2A receptors) (Bajaj et al., 2014), whereas triheteromeric GluN2A receptors are composed of 2 GluN1 and 2 GluN2 and/or GluN3 subunits (GluN1/GluN2A/GluN2B, GluN1/GluN2A/GluN2C, and GluN1/GluN2A/GluN3 receptors) (Hansen et al., 2014). GluN1/GluN2A/GluN2B receptors may be one of the most abundant NMDARs in the forebrain of adult animals (Chazot and Stephenson, 1997); previous studies have shown that GluN1/GluN2A/GluN2B receptors account for 15%–40% of the NMDARs in the hippocampus of rats (Al-Hallaq et al., 2007). Growing evidence has shown that the coexistence of diheteromers and triheteromers within a single cell or at a single synapse contributes to the functional diversity of the postsynaptic response (Shipton and Paulsen, 2014). However, some past studies have revealed that there are differences in the functional and pharmacological features and characteristics between diheteromeric GluN1/GluN2A receptors and triheteromeric GluN1/2A/2B receptors. For example, GluN1/GluN2A receptors exhibit faster deactivation, faster desensitization recovery, and higher channel opening probability (PO; highly sensitive to Mg^2+^ blockade and Ca^2+^ permeability) than other diheteromeric GluN2-containing NMDARs (Krupp et al., 1996; Vicini et al., 1998; Chen et al., 1999).

The time constant for glutamate deactivation of GluN1/GluN2A/GluN2B receptors is 5.5 times faster than that of GluN1/GluN2B receptors, but it is 1.8 times slower than that of GluN1/GluN2A receptors (Hansen et al., 2014). These electrophysiological properties enable GluN2A subunits to regulate synaptic activity more flexibly. In addition, a distinctive intra- and inter-subunit interface was found in GluN1/2A/2B NMDARs triheteromers, whereas it was absent in GluN1/GluN2A NMDARs diheteromers (Lü et al., 2017).

### GluN2A Subunit: Binding Partners

**Figure 5.**
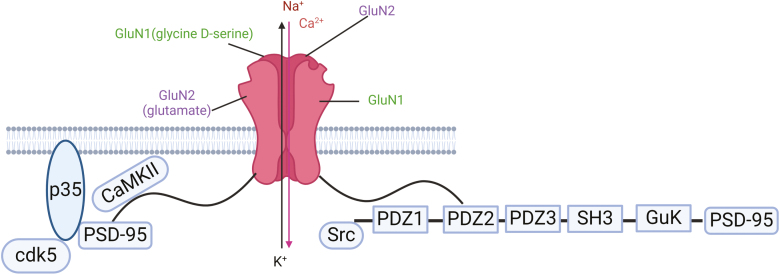
Graphic representation of GluN2A interactors at CTD. The domain structure of PSD-95 includes 3 PDZ domains (PDZ1, PDZ2, and PDZ3), 1 SH3 domain and a guanylate kinase homology (GuK) domain. PSD-95 is the most widely studied and it can form a complex in synaptosomes with p35 and cyclin dependent kinase-5 (cdk5). In addition, the SH2 domain of Src interacts with the N-terminal region of PSD-95 (43-57). The figure was generated using BioRender (Agreement number: MQ272TFCJW).

The GluN2A subunit has an intracellular CTD associated with intracellular transport and signal transduction in the receptor. The CTD of the GluN2A subunit interacts with a variety of intracellular proteins, generating a multiprotein complex in neurons involved in NMDAR trafficking, clustering, localization, and signaling (Collingridge et al., 2004; Kim and Sheng, 2004). There are 4 GluN2A CTD interacting partners: scaffolding proteins (PSD-95, PSD-93, SAP102, SAP97, Rph3A, and FRMPD2), synapse-to-nuclear messengers (RNF10, AIDA-1d, and ERK), protein kinases (Ca^2+^/calmodulin-dependent protein kinase II [CaMKII] and CdK5), and other proteins (GEFs, and Ca2+/Calmodulin complexes) (Franchini et al., 2020). For example, the last 3 amino acid sequences (1461–1464) in the C terminus of GluN2A bind directly to several neuronal scaffolding proteins, such as members of membrane-associated guanylate kinase: postsynaptic density protein-93 (PSD-93), postsynaptic density protein-95 (PSD-95), synapse-associated protein 97 (SAP97), and synapse-associated protein 102 (SAP102) (Lim et al., 2002; Howard et al., 2010), which are responsible for GluN2A-containing NMDARs retention in PSD. Among these scaffolding proteins, PSD-95 is the most widely studied, and it can form complexes in synaptosomes with p35 and cyclin-dependent kinase-5 (cdk5) (Morabito et al., 2004). The domain structure of PSD-95 includes 3 PDZ domains (PDZ1, PDZ2, and PDZ3), 1 SH3 domain, and a guanylate kinase homology domain (Figure 5) (Cho et al., 1992), each of which mediates protein–protein interactions (Kim and Sheng, 2004). Kalia et al. demonstrated that amino acids 43–57 within PSD-95, from the N terminus to the PDZ1 domain, interact directly with the SH2 domain of Src and suppress the tyrosine kinase activity of Src (Kalia et al., 2006). In addition, the 1349–1464 amino acid sequence at the C terminus of GluN2A is responsible for the binding of CaMKII (Gardoni et al., 1999).

## ROLES OF GluN2A IN DEPRESSION

Depression is the most common mental disorder, affecting 1 in every 5 people in their lifetime, and is the leading cause of disability worldwide. The COVID-19 pandemic has added a marked depressive burden to the situation (Pérez-Cano et al., 2020). Data from 204 countries imply that the COVID-19 pandemic and associated lockdowns led to a 27.6% increase in MDD cases throughout 2020 (Santomauro et al., 2021). Depressive disorders are disabling conditions that occur at all ages and are characterized by depressed moods, social isolation, and anhedonia. It is well known that a combination of genetic predisposition and life events has been recognized to contribute to the development of depression. Presently, the treatment of depressive disorders involves pharmacological and psychotherapeutic interventions. Nonpharmacological approaches are as effective as pharmacological therapies for mild-to-moderate depression. However, approximately 20%–40% of patients with a major depressive episode do not exhibit a clinical response to current treatment with antidepressants, or patients with depression often succumb to an immediate relapse of depression after drug withdrawal, even in cases of effective antidepressant reagents (Skolnick, 2002). Therefore, the ineffective treatment of MDD requires immediate improvement in regard to the therapeutic approach by developing clinically useful and easily accessible antidepressants with high accuracy. Several animal model studies have revealed the biological mechanisms of depression, including altered neurotransmission, inflammatory responses, hypothalamic-pituitary-adrenal axis abnormalities, reduced neuroplasticity, and microbiota-gut-brain axis dysfunction (Nedic Erjavec et al., 2021; Zhang et al., 2022). Nevertheless, significant progress (the development of novel rapid-acting antidepressants) has been made in the last few decades based on the aforementioned biological mechanisms; however, the precise mechanisms associated with the pathogenesis of depression are yet to be completely understood.

### GluN2A Subunit and Synaptic Plasticity

It is generally believed that the dramatic changes across postnatal development at the level of the GluN2B-GluN2A subunit “switch” (a shift from reliance on GluN2B-containing receptors to reliance on GluN2A-containing receptors) are in response to neuronal activity and sensory experiences during development (Matta et al., 2011). GluN2A-containing NMDAR has been shown to exhibit specific channel properties that generate distinct postsynaptic calcium dynamics. In view of the binding of the GluN2A CTD to the variety of synaptic proteins mentioned above, GluN2A confers NMDAR-specific electrophysiological properties, making these receptors versatile modulators of synaptic activity. In addition, multiple studies have indicated that GluN2A plays important roles in synaptic plasticity; for example, Sakimura et al. indicated that long-term potentiation (LTP) induced by high-frequency stimulation in the hippocampal CA1 and juvenile superior colliculus was significantly reduced after targeted disruption of the GluN2A gene in mice (Sakimura et al., 1995). However, Li et al. found that elevated synaptic GluN2A-NMDAR impaired long-term synaptic plasticity in mouse hippocampal CA1 neurons (Li et al., 2022). By considering these conflicting results, the exact mechanism of action of GluN2A on synaptic plasticity remains elusive. Changes in brain plasticity may be the primary cause of depression. Based on the aforementioned analysis, we can speculate that GluN2A is involved in the pathogenesis of depression; however, the underlying mechanisms remain unknown.

Recent studies have indicated that changes in brain plasticity may be the primary cause of depression (Ménard et al., 2016). Environmental events and other risk factors have been shown to contribute to depression by disrupting the functional and structural connections of neural circuits that underlie mood (Duman et al., 2016). For example, postmortem studies have reported that synapse number and function are decreased in the dorsolateral prefrontal cortex of patients with depression (Drevets, 2000). Moreover, animal studies, such as a depression model, have confirmed that chronic stress causes neuronal atrophy and synaptic loss in the hippocampus (Shi et al., 2022; Guan et al., 2023). Therefore, changes in neural plasticity induced by stress and other negative stimuli may play a significant role in the onset and development of depression. There is evidence that NMDARs are critical for neuroplasticity, and stressors induce excessive NMDAR activity that could result in the pathology of depression (Marsden, 2011). GluN2A, a major subunit that is highly expressed in the synaptic cleft, plays a significant role in neuroprotection and neuroplastic enhancement (Table 1). As aforementioned, the GluN2A subunit is involved in neuroinflammation-related depression, indicating that LPS induces glutamate excitotoxicity via excessive NMDAR activity, including GluN2A overexpression. Recognizing this, we speculate that stress-induced GluN2A overexpression leads to synaptic plasticity impairment, thereby inducing depressive states.

**Table 1.** GluN2A-mediated Synaptic Plasticity Deficits

Animal models	GluN2A antagonist	Main findings	Mechanisms	References
*Fmr1*^*−/y*^ mice	TCN‐201	Impaired synaptic plasticity was restored by pharmacologically inhibiting GluN2A‐containing NMDA receptors in *Fmr1*^*−/y*^ mice	Pre‐incubation hippocampal slices with selective GluN2A antagonist TCN‐201 not only completely rescued impaired LTP but also reduced enhanced mGluR‐LTD observed in *Fmr1*^*−/y*^ mouse	(Lundbye et al., 2018)
The NAc from male C57BL/6 mice	NVP-AAM077	Dopamine produced LTP of NMDA responses, which occluded LTD induced by HFS of glutamatergic fibers	LTD induced by dopamine and HFS required GluN2A	(Chergui, 2011)
MK-801-induced depressive-like behavior in rats	NVP-AAM077	Antagonists selective for GluN2A (NVP-AAM077) possessed antidepressant-like activity	Enhanced release of glutamate and 5-HT induced by NVP-AAM077 and its action on AMPARs contributed to release of BDNF and subsequently increased synaptogenesis and synaptic plasticity	(Jiménez-Sánchez et al., 2014)
*Grin2a*^fl/fl^ mice stereotaxically injected with high-titer rAAV1-Cre:GFP viral stock	GluN2A knockout mice	GluN2A subunit was strictly necessary for either non-ionotropic or ionotropic LTD	NMDAR-mediated LTD was independent of GluN2A	(Wong and Gray, 2018)
Adult male C57BL/6 mice	NVP-AAM077	NVP-AAM077 had no significant effect on induction of synaptic depression after potentiation	Persistence of LTP could be disrupted by LFS; this synaptic depression was time dependent	(Xue et al., 2021)
Forebrain specific GluN2A overexpression TG mice	GluN2A overexpression	NMDAR-dependent LTD were impaired at T-LA pathway in GluN2A TG mice	GluN2A overexpression maintained normal basal synaptic transmission and NMDAR-dependent LTP at T-LA synapses in TG mice, but they were deficit in NMDAR-dependent LTD	(Wang et al., 2021b)
Adult male Sprague Dawley rats	Slow-onset increase in GluN2A	CORT-mediated slow-onset increase in GluN2A in hippocampal synapses could be a homeostatic mechanism to normalize synaptic plasticity following fast-onset stress-induced facilitation	CORT potentiated evoked NMDAR-mediated synaptic responses (LTP and LTD) within 30 min, while a slow-onset increase in synaptic expression of GluN2A-containing NMDARs and bidirectional plasticity was observed 1-2 hours after CORT treatment	(Tse et al., 2011)

Abbreviations: LFS, low-frequency stimulus; TG, transgenic; T-LA, thalamus-lateral amygdala; LTP, long-term potentiation; NAc, nucleus accumbens; LTD, long-term depression; mGluR, metabotropic glutamate receptor; CORT, corticosterone.

Lundbye et al. provided experimental evidence to show that dampening the elevated levels of GluN2A‐containing NMDARs in *Fmr‐1 knock‐out* (*Fmr1*^*−/y*^) mice restored hyperexcitability of the neural circuitry to normal‐like level of brain activity (Lundbye et al., 2018). They found impaired NMDAR‐dependent LTP and elevated metabotropic glutamatergic receptor subtype 5‐dependent LTD in *Fmr1*^*−/y*^ mice, while pre-incubation hippocampal slices with the selective GluN2A antagonist TCN-201 not only completely rescued impaired LTP but also reduced the enhanced mGluR-LTD observed in *Fmr1*^*−/y*^ mice, suggesting the activity of GluN2A can up- or downscale synaptic strength by changing the thresholds of LTP and LTD. Similarly, Chergui et al. (Chergui, 2011) have shown that GluN2A plays a critical role in dopamine-induced and high-frequency stimulation (HFS)-induced synaptic plasticity. They showed that dopamine required GluN2A to inhibit NMDAR-mediated synaptic transmission in the nucleus accumbens (NAc) of mice, which occluded LTD induced by the HFS of glutamatergic fibers. Thus, we speculate that the long-lasting depression induced by dopamine occludes HFS-LTD, and the depressant action of dopamine on NMDAR function in the NAc of mice might involve a direct protein–protein interaction between the dopamine receptors and GluN2A, as demonstrated by Lee et al. (Lee et al., 2002). In support of this view, Shen et al. (Shen et al., 2021) assessed the effects and mechanism of action of *Polygonatum sibiricum* polysaccharide (PSP) on depression-like behaviors. The results showed that PSP administration prevented LPS-induced synaptic damage through the upregulation of hippocampal GluA1 and GluA2 (subunits of the AMPA receptor) but decreased hippocampal GluN2A expression. Interestingly, Jiménez-Sánchez et al. found an enhanced release of glutamate and 5-HT induced by antagonists selective for GluN2A (NVP-AAM077), and its action on AMPARs contributed to the release of brain-derived neurotrophic factor (BDNF) and subsequently increased synaptogenesis and synaptic plasticity, suggesting an effective antidepressant action (Jiménez-Sánchez et al., 2014). However, the authors raised concerns that NVP-AAM077 might lead to cognitive deficits, including impaired working memory, disruptions in regard to spatial learning, and increased aberrant gamma activity (Hu et al., 2009; Smith et al., 2011; Kocsis, 2012). Therefore, further research is required to confirm the potential antidepressant efficacy of selective GluN2A receptor antagonists and cognitive impairment after chronic stress.

Despite the positive results in rodent research, studies testing this hypothesis have yielded inconsistent and often contradictory results. For example, Wong et al. (Wong and Gray, 2018) found that neither the GluN2A nor the GluN2B subunit was strictly necessary for either non-ionotropic or ionotropic LTD. They explored the hypothesis that different NMDAR subunits dictate the rules of synaptic plasticity, especially the roles of GluN2A in LTD, using a single-neuron genetic approach to delete NMDAR subunits in conditional knockout mice (Wong and Gray, 2018). Researchers have concluded that this contradictory result is likely due to issues with GluN2A subunit-selective pharmacology and a high proportion of synaptic triheteromeric NMDARs (Wong and Gray, 2018). In line with the aforementioned results, Xue et al. found that the GluN2A-selective antagonist NVP-AAM077 did not affect the induction of synaptic depression after potentiation in the anterior cingulate cortex of mice by a low-frequency stimulus (1 Hz, 15 minutes) (Xue et al., 2021) and hypothesized that synaptic potentiation or depression may employ different mechanisms in different brain regions. It is also intriguing that forebrain-specific GluN2A overexpression maintains normal basal synaptic transmission and NMDAR-dependent LTP at thalamus-lateral amygdala (T-LA) synapses in transgenic (TG) mice, but deficits in NMDAR-dependent LTD were observed in in vitro electrophysiological data (Wang et al., 2021b). In comparison, knowledge of the role of GluN2A in LTP and LTD is inconsistent (Lundbye et al., 2018). We speculate that this contradictory result may be linked to the animal models used and different types of brain tissues. Moreover, we are left to wonder why GluN2A overexpression does not affect LTP in synaptic plasticity at T-LA synapses. Thus, we deduced that GluN2A overexpression allows more Ca^2+^ to cross the postsynaptic membrane and contributes to the increased activity of the alpha subunit of CamKII (αCaMKII). αCaMKII is the most abundant synaptic protein in forebrain structures, such as the hippocampus, while its expression begins postnatally in glutamatergic excitatory neurons and it has been shown to constitute the major PSD protein (Kennedy, 1997; Schulman, 2004). αCaMKII is activated by synaptic transmission-mediated calcium influx, and its subsequent phosphorylation is central to synaptic plasticity. The only plausible explanation is that the effects of αCaMKII activity on synaptic strength may be counteracted with the inhibitory effect of αCaMKII phosphorylation on other substrates (Lu et al., 2010), but the specific mechanisms are needed to be elucidated further.

In summary, these findings show here that GluN2A plays an important role in synaptic plasticity both in physiological and pathological conditions. Although GluN2A has been intensively studied in the last decades in animal models, the impact of the reduction of GluN2A expression on synaptic plasticity in human studies has not been fully understood. Thus, more experiments should be performed to elucidate GluN2A properties in human trials.

### Roles of GluN2A in Neuroinflammation-Related Depression

**Figure 6.**
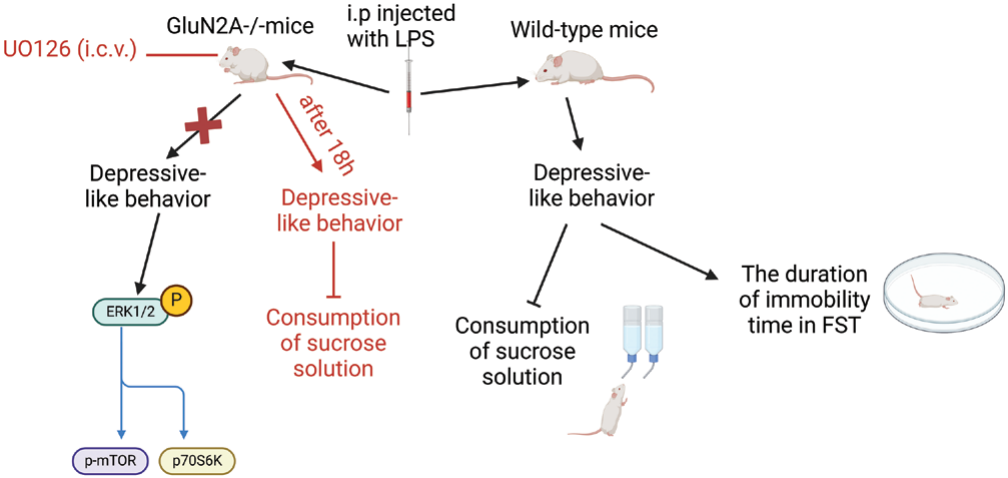
The lack of the GluN2A subunit abolished depressive outcomes when challenged with LPS by sustaining mTOR pathway activity and preserving synaptic stability. Mice lacking GluN2A subunit (GluN2A^−/−^ mice) did not display changes in depressive-like behavior including the consumption of sucrose solution and the duration of immobility time in forced swim test (FST) after the administration of LPS compared with LPS-treated wild-type mice. Interestingly, LPS treatment increased synaptic levels of downstream effectors (p-mTOR and total p70S6K) of mTOR activity in the hippocampus of GluN2A^−/−^ mice. Most importantly, 18 hours after extracellular signal-regulated kinase (ERK) inhibitor (UO126) administration in LPS-treated GluN2A^−/−^ mice, UO126 was found to significantly decrease the consumption of sucrose solution compared with the vehicle-infused GluN2A^−/−^ mice (Francija et al., 2022).The figure was generated using BioRender (Agreement number: XC272TFGUM).

Inflammation plays a key role in initiating depression in some individuals, and depression has inflammatory consequences (Kiecolt-Glaser et al., 2015). A recent study by Francija et al. showed that GluN2A-extracellular signal-regulating kinase (ERK)-mechanistic target of rapamycin (mTOR) signaling is a vulnerability factor for inflammation-related depressive behavior (Francija et al., 2022). They found that mice lacking the GluN2A subunit (GluN2A^−/−^ mice) did not display changes in locomotor activity and depressive-like behavior, including the consumption of sucrose solution and the duration of immobility time in the forced swim test after the immune challenge (administration of LPS 0.83 mg/kg) compared with LPS-treated WT mice (Francija et al., 2022). Further research showed LPS treatment produced no effect on the activity of the mTOR pathway in the hippocampus and prefrontal cortex (PFC) of GluN2A^−/−^ mice by measuring synaptic levels of upstream regulators (ERK, protein kinase B (Akt), and GSK3β, as well as their active, phosphorylated forms). Interestingly, LPS treatment increased the synaptic levels of the downstream effectors (p-mTOR and total p70S6K) of mTOR activity in the hippocampus of GluN2A^−/−^ mice, whereas PFC treatment did not affect p-mTOR or total p70S6K levels (Francija et al., 2022). Thus, we can speculate that sustained mTOR activity, especially increased synaptic levels of downstream effectors (p-mTOR and total p70S6K) in the hippocampus, plays an important role in the resilience of GluN2A^−/−^ mice to depression, which also confirms mTOR-mediated synaptic function (Zhou et al., 2023). In addition, the authors also assessed the changes in the levels of 2 important synaptic markers, GluA1 and PSD-95. They found that LPS treatment did not affect GluA1 or PSD-95 levels in the hippocampus of GluN2A^−/−^ mice, whereas LPS decreased GluA1 levels in the PFC. Most importantly, 18 hours after ERK inhibitor (UO126) administration in LPS-treated GluN2A^−/−^ mice, UO126 significantly decreased the consumption of sucrose solution compared with vehicle-infused GluN2A^−/−^ mice, which was not observed 4 hours after inhibitor administration, indicating that the resilience of GluN2A^−/−^ mice to LPS-induced depressive-like behavior was abolished by the inhibition of ERK (Francija et al., 2022). In summary, these results showed that the lack of the GluN2A subunit abolished depressive outcomes when challenged with LPS by sustaining mTOR pathway activity and preserving synaptic stability and that the resistance of GluN2A knockouts to LPS-induced depressive-like behavior was ERK dependent (Figure 6). Therefore, we believe that this knowledge is vital for the development of selective antagonists targeting GluN2A subunits using pharmacological and molecular methods. Thus, GluN2A NMDAR antagonists have the potential to be developed as neuroprotective drugs with optimal therapeutic effects.

Similarly, another study reported that the absence of the GluN2A subunit fully abrogated the LPS-induced depressive phenotype in mice (Francija et al., 2019). Specifically, although LPS treatment increased immobility and reduced the consumption of sucrose solution in WT mice, it did not affect depressive-like behavior in GluN2A KO mice, suggesting GluN2A KO mice displayed resistance to depressive-like behavior upon LPS treatment (Francija et al., 2019). In addition, there was no significant difference in cytokine levels (IL-6) in the hippocampus and PFC between LPS-treated GluN2A KO mice and controls (VEH) (Francija et al., 2019). Interestingly, in GluN2A KO mice, LPS significantly increased BDNF levels in hippocampus and increased the levels of pro-BDNF both in hippocampus and PFC. Furthermore, LPS treatment increased all neural cell adhesion molecule (NCAM) isoforms (120/140/180 kDa) in PFC of GluN2A KO mice, while it had no effects on the levels of hippocampal NCAM isoforms (Francija et al., 2019), indicating GluN2A KO mice potentiated synaptic stabilization through NCAM in the PFC upon LPS treatment. Finally, the authors found that LPS treatment increased GluN2A subunit levels in the PFC of WT mice but did not affect this NMDAR subunit in the hippocampus (Francija et al., 2019). These results suggest that the resilience of GluN2A KO mice to depressive-like behavior upon LPS treatment might be attributed to increased proBDNF levels and preserved polysialylated-NCAM in the hippocampus and PFC, as well as elevated NCAM in the PFC. There is also evidence that PSP ameliorates LPS-induced depression-like behavior by reducing the expression of GluN2A in the hippocampus of mice (Francija et al., 2019). These aforementioned results indicated that the GluN2A subunit is involved in neuroinflammation-related depression, whereas its absence abolishes the LPS-induced depressive phenotype. It has been suggested that LPS provokes tryptophan–kynurenine metabolic pathway dysregulation and results in the synthesis of the neurotoxic NMDA glutamate agonists quinolinic acid and 3-hydroxykynurenine, thereby enhancing oxidative stress and contributing to depressive-like behavior (O’Connor et al., 2009).

In summary, the absence of the GluN2A subunit abolishes the LPS-induced depressive outcomes (Table 2), and this hypothesis identifies possibilities for the development of new types of antidepressants, which will hopefully also improve their efficacy. Therefore, this review summarizes recent findings regarding the pathophysiological role of GluN2A in depression. Moreover, a recent study showed that GluN2A mediates ketamine-triggered rapid antidepressant-like responses (Su et al., 2023). Thus, GluN2A is a promising target for the development of novel antidepressant drugs.

**Table 2.** Absence of GluN2A Subunit Abolishes LPS-Induced Depressive Outcomes

Animal models	GluN2A expression	Main findings	Mechanisms	References
LPS-induced model of depression	GluN2A KO mice (downregulation)	GluN2A-ERK-mTOR pathway conferred a vulnerability to LPS-induced depressive-like behavior	Lacking GluN2A subunit abolished depressive outcomes when challenged with LPS by sustaining mTOR pathway activity and preserving synaptic stability	(Francija et al., 2022)
LPS-induced depressive-like behavior in mice	GluN2A KO mice (downregulation)	Absence of GluN2A abolished LPS-induced depressive phenotype	Resilience of GluN2A KO mice to depressive-like behavior upon LPS might be attributed to increased proBDNF levels and preserved PSA-NCAM in hippocampus and PFC, as well as elevated NCAM in PFC	(Francija et al., 2019)
LPS-induced depressive-like behavior in mice	Downregulation	Expression of GluN2A was reduced in hippocampus of LPS-induced mice	Polygonatum sibiricum polysaccharide (PSP) prevented depression-like behaviors by reducing GluN2A expression	(Shen et al., 2021)
LPS-induced depressive-like behavior in mice	Upregulation	Protein levels of GluN2A were increased in hypothalamus of LPS-induced mice	Elevated expression of EPHB2-GluN2A-AKT cascade led to impairment of synaptic plasticity and ultimately depressive-like behavior	(Wu et al., 2019)
LPS-induced depressive-like behavior in mice	Downregulation	Ginsenoside Rg1 reduced increase of hippocampal GluN2A induced by LPS	Protective effects of ginsenoside Rg1 against depression-like behaviors in mice, likely via promoting synaptic proteins and reducing GluN2A in hippocampus	(Zhang et al., 2021b)

Abbreviations: EPHB2, Ephrin type-B receptor 2; PSA, polysialylated.

## GluN2A MEDIATES KETAMINE-INDUCED RAPID ANTIDEPRESSANT-LIKE RESPONSES

**Figure 7.**
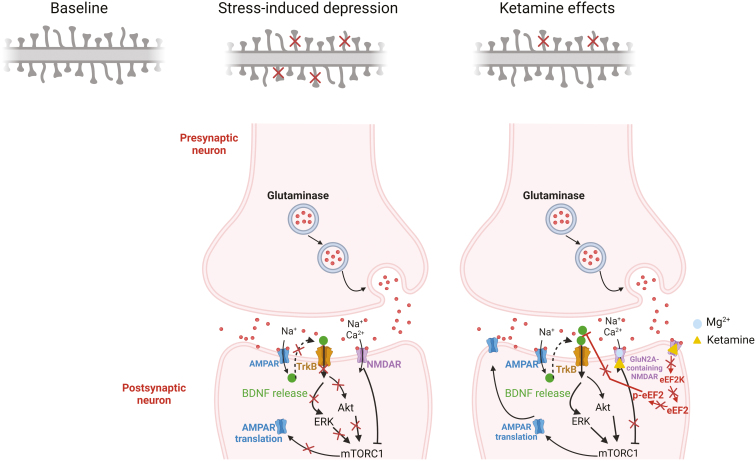
Schematic of proposed mechanisms of ketamine’s antidepressant effect. Evoked released glutamate binds to and activates post-synaptic AMPARs resulting in enhanced BDNF release, activation of the tropomyosin receptor kinase B (TrkB) receptor and subsequently promotion of protein synthesis via the activation of the mechanistic target of rapamycin complex 1 (mTORC1) (Yang et al., 2013). In addition, ketamine blocks NMDAR-mediated spontaneous neurotransmission, which results in the inhibition of the eukaryotic elongation factor 2 kinase (eEF2K) activity, thus preventing phosphorylation of its eEF2 substrate. This effect subsequently leads to an enhancement of BDNF translation (Autry et al., 2011). The figure was generated using BioRender (Agreement number: ZA272TEVHR).

Severe depression affects approximately 16% of the world’s population at some point in their lives, and it significantly reduces their quality of life (Kessler et al., 2003). Despite existing monoaminergic-based pharmacotherapies, most patients require prolonged administration (weeks, if not months) for clinical improvement. Placebo-controlled trials have provided strong evidence that a single sub-anesthetic dose of the dissociative anesthetic ketamine (an NMDAR antagonist) induces rapid (within hours) and sustained antidepressant actions (lasting up to 7 days) in treatment-resistant patients (Berman et al., 2000; Zarate et al., 2006). Despite the promise of ketamine, key challenges remain, including how to maintain the response, concerns regarding short- and long-term side effects, and the potential for abuse (Short et al., 2018). Recent preclinical research has focused on the role of GluN2A in mediating ketamine-induced rapid antidepressant-like responses in an effort to develop novel pharmacotherapies that mimic the antidepressant actions of ketamine but lack the undesirable effects of ketamine (Su et al., 2023). Ketamine is a racemic compound composed of equal amounts of (*R*)-ketamine and (*S*)-ketamine, with the (*S*)-enantiomer having a greater affinity for NMDAR. (*S*)-Ketamine has been shown to be effective as an antidepressant when administered via both i.v. and intranasal routes (Singh et al., 2016; Daly et al., 2018). In addition, intranasal (*S*)-ketamine decreases suicidal ideation in patients with depression (Canuso et al., 2018). In contrast, limited clinical research exists on the antidepressant effects of (*R*)-ketamine’s antidepressant effects (Leal et al., 2021). Overall, the pharmacological target deconvolution of ketamine will provide insights critical to the development of new pharmacotherapies that possess the desired clinical effects of ketamine. Su et al. were the first to report GluN2A-mediated ketamine-triggered rapid antidepressant-like responses (Su et al., 2023). Specifically, the GluN2A, but not GluN2B, is involved in the antidepressant-like behavioral effects of broad NMDAR channel blockers, such as ketamine, by increasing the intrinsic excitability of hippocampal principal neurons, while the acute antidepressant-like effects of ketamine were abolished in mice that lacked GluN2A (Su et al., 2023). These putative neurobiological mechanisms underlying GluN2A-mediated rapid-acting antidepressant effects may be used to develop the next-generation rapid-acting antidepressants that lack the side effects or abuse potential of ketamine. Further supporting this hypothesis, Zanos et al. found that the antidepressant-like actions of ketamine were mediated through GluN2A activity (the GluN2A NMDAR-positive modulator GNE-5729 at a dose of 3 mg/kg) in the hippocampus of CSDS-induced mice, whereas the GluN2A subunit-selective NMDAR antagonist PEAQX (at doses of either 5 or 30 mg/kg) prevented the antidepressant-like effects of ketamine (Zanos et al., 2023). Collectively, these results indicated that ketamine requires and potentially acts downstream via GluN2A activation to exert its antidepressant behavioral actions (Figure 7).

However, the evidence on the role of GluN2A in ketamine production is controversial. For example, in a study conducted by Treccani et al. (Treccani et al., 2019), apical dendritic spine deficits in the CA1 pyramidal neurons of Flinders Sensitive Line rats were completely restored 1 hour after injection with ketamine but had no effect on apical and basal dendritic arborization. The same authors showed that the ketamine-induced increase in dendritic spine density in rats subjected to Flinders Sensitive Line was associated with a reduction in the synaptic levels of GluN2A and cofilin phosphorylation, together with increased homer3 protein expression levels (Treccani et al., 2019). This result indicates that the rapid actions of ketamine are linked to the downregulation of GluN2A, increased homer3 levels, and normalization of cofilin activity. Hasegawa et al. found that ketamine attenuated social impairment induced by social defeat stress in juveniles via the activation of AMPARs in the hippocampus of mice, whereas a selective AMPAR antagonist (NBQX) inhibited the attenuating effect of ketamine (Hasegawa et al., 2019). Importantly, the authors demonstrated that there were no significant changes seen in the ratios of GluN2A at Tyr^1325^ and the levels of total GluN2A protein in the hippocampus or PFC of GluN2A KO mice (Hasegawa et al., 2019), suggesting that the NMDAR GluN2A subunit may not be involved in the expression of impaired social behaviors.

**Figure 8.**
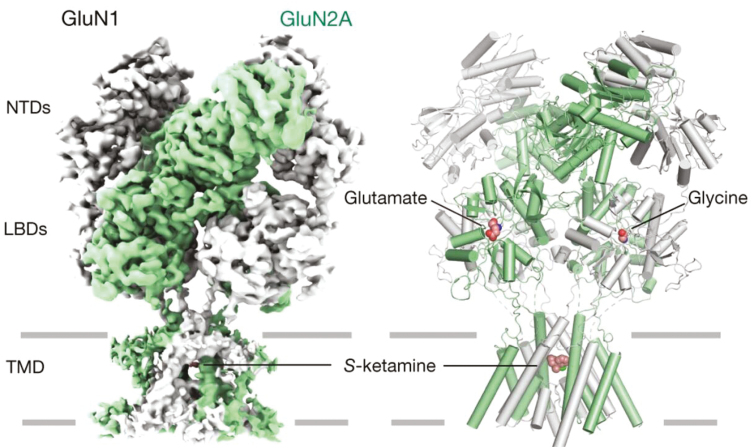
Cryo-EM densities (left) and structural models (right) of human GluN1–GluN2A receptors. GluN2A is represented in green with glutamate presented in the clefts, while the GluN1 subunits are represented in grey with glycine captured in the LBDs. (*S*)-ketamine was captured within the TMD in GluN1-GluN2A NMDARs. The figure was adapted from Zhang et al. (Zhang et al., 2021a).

Consistent with this, Liu et al. demonstrated that repeated administration of (*S*)-ketamine for 7 consecutive days reversed CUMS-induced depression-like behaviors and synaptic ultrastructural alterations in the hippocampus of mice via the upregulation of GluN1 and GluR1 levels and downregulation of GluN2B levels (Liu et al., 2023), whereas there was no significant effect of CUMS or ketamine treatment on GluN2A subunit expression. Remarkably, Lecointre et al. showed age-dependent alterations in the GluN2A developmental profile and adult behavior in postnatal ketamine-treated mice (Lecointre et al., 2015). Specifically, ketamine increased GluN2A mRNA levels at the postnatal age in injection (P2)-treated mice without any changes in proteins, whereas it reduced both the GluN2A mRNA and protein levels in P5-treated mice (Lecointre et al., 2015). Interestingly, there were no obvious changes in GluN2A expression in the P10-treated mice. This finding indicates that ketamine impairs the developmental profile of the cortical NMDAR subunit and delays the synaptic targeting of GluN2A-containing NMDARs. In brief, these findings extend the existing literature that indicates that GluN2A is involved in the effects of ketamine on ameliorating symptoms of treatment-resistant depression and may have implications for understanding the cellular and molecular mechanisms underlying the antidepressant effects of ketamine (Figure 8).

Studies in rodents have suggested that the GluN2A subunit is involved in the sustained antidepressant-like effect of ketamine and that the GluN2A subunit may be a significant factor in this persistence. In the present review, we did not observe GluN2A-mediated antidepressant-like effect of ketamine on patients with depression, which is a limitation. Despite the promise of the involvement of GluN2A in the antidepressant-like effect of ketamine, some problems, including how to maintain response, the adverse effect, and the potential for abuse, remain. These side effects are directly related to dose amount. Certainly, more work is needed to address these concerns.

## CONCLUSIONS

To the best of our knowledge, this is the first systematic review of the literature on the role of GluN2A in depression. Based on the evidence presented here, it is apparent that GluN2A-containing NMDARs are involved in the pathogenesis of depression, including inflammatory responses and synaptic plasticity. Therefore, the role of GluN2A in depression has attracted increasing attention. In this review, we found that the GluN2A subunit is critical in neuroinflammation-related depression, and its absence abolished the LPS-induced depressive phenotype (Francija et al., 2019, 2022), indicating that GluN2A KO mice displayed resilience to depressive-like behavior upon LPS treatment. These results present a new avenue for specific inhibition of GluN2A NMDAR in the treatment of neuroinflammation-related depression. Because the specific inhibition of GluN2A is reasonably effective in attenuating the symptoms of depression, it is equally effective in attenuating the impairment of synaptic plasticity, which also plays a significant role in the onset and development of depression. There is now evidence that pharmacologically inhibiting GluN2A‐containing NMDARs restored the hyperexcitability of the neural circuitry to normal‐like level of brain activity and impaired synaptic plasticity via rescuing impaired LTP and reducing the enhanced mGluR‐LTD in hippocampus of *Fmr1*^*−/y*^ mice (Lundbye et al., 2018). Thus, changes in GluN2A expression were associated with complex phenotypes that led to complex psychiatric disorders, including the occurrence of depression. However, other past studies have reported inconsistent results. For example, Wong et al. used a single-neuron genetic approach to delete NMDAR subunits in conditional KO mice and confirmed that the GluN2A subunit is necessary for both non-ionotropic and ionotropic LTD (Wong and Gray, 2018). We speculate that these conflicting results regarding the role of GluN2A in LTD are likely due to issues with the GluN2A subunit-selective pharmacology (e.g., NVP-AAM077, a GluN2A “selective antagonist,” is only fivefold selective over GluN2B), complex effects on glutamate affinity, and incomplete blockade. In addition, the modestly responsive to GluN2A subunit-selective pharmacology of a high proportion of synaptic triheteromeric NMDARs and the GluN2A KO mice-induced altered network activity were attributed to these differences.

In this review, we have also discussed the role of GluN2A in ketamine’s antidepressant actions. Ketamine is a glutamate NMDAR antagonist used to treat MDD via single or repeated infusions. It has been suggested that the loss of GluN2A in adult mouse brains elicits robust antidepressant-like responses, whereas the antidepressant-like effects are mediated through GluN2A in order to increase the intrinsic excitability of hippocampal principal neurons (Su et al., 2023). Nevertheless, the efficacy, safety, and tolerability of maintenance ketamine treatment for depression have received considerable attention in recent years. For example, a chronically depressed patient administered a course of 6 alternate-day IV ketamine infusions (0.5 mg/kg) remained in remission for 26 days but relapsed after a further 3 days (Messer et al., 2010). The potentially harmful consequences of prolonged ketamine use include ulcerative cystitis, liver injury, neurocognitive impairment, and addiction (Smith-Apeldoorn et al., 2022). From a positive perspective, Zanos et al. concluded that the metabolism of ketamine to (2R,6R)-hydroxynorketamine is essential for its antidepressant effects and this compound lacked ketamine-related side effects (Zanos et al., 2016). They also established that the antidepressant actions of (2R,6R)-hydroxynorketamine were NMDAR inhibition independent but involved early and sustained AMPAR activation (Zanos et al., 2016). Therefore, with the growing interest in ketamine as a treatment for depression, as well as the increasing use of repeated dosing in both clinical and research settings, it is necessary to further explore and systematically assess the clinical applicability of maintenance ketamine treatment.

In conclusion, the findings of the present review suggest that the GluN2A subunit is involved in the pathogenesis of depression and that the GluN2A subunit may be a significant target for antidepressant therapy. Furthermore, these findings support the expansion of research into the role of GluN2A in ketamine treatment as a novel antidepressant for use in a clinical setting.

### Limitations

These findings further confirm the role of GluN2A-containing NMDARs in the rapid antidepressant action of ketamine. Despite its antidepressant-like effects, its safety and tolerability should not be overlooked. Concerning intranasal (*S*)-ketamine administration, most adverse events are of mild or moderate severity, such as dizziness, dissociation, dysgeusia, vertigo, and nausea (Canuso et al., 2018; Popova et al., 2019), while adverse events usually occur with very high doses that are administered for prolonged periods of time (Zhu et al., 2016). Therefore, regarding the role of GluN2A in the rapid antidepressant-like effect of ketamine, specific inhibition of GluN2A NMDAR may be developed to eliminate the side effects of ketamine and improve safe and effective innovative treatments for depression. However, further research is required to confirm this hypothesis. Most candidate molecules for GluN2A antagonists have been studied only in rodent models of depression without successful extension to humans. It is therefore essential to continue research into the safety profile of specific inhibition of GluN2A to identify the optimal dosage in patients with depression. Furthermore, large sample sizes are needed to support the potential benefit of GluN2A antagonists for treatment of depression.

## Data Availability

All the data extracted from included original articles are available in PubMed or Web of Science. This review was conducted without previous registration, and no protocol document was prepared.
